# Impact of COVID-19 pandemic on Vascular Surgery Unit activity in Central Romania

**DOI:** 10.3389/fsurg.2022.883935

**Published:** 2022-08-23

**Authors:** Arbănași Emil-Marian, Kaller Reka, Mureșan Vasile Adrian, Voidăzan Septimiu, Arbănași Eliza-Mihaela, Russu Eliza

**Affiliations:** ^1^ Clinic of Vascular Surgery, Mureș County Emergency Hospital, Târgu-Mureș, Romania; ^2^ Department of Surgery, University of Medicine, Pharmacy, Science and Technology “George Emil Palade” of Târgu-Mureș, Târgu-Mureș, Romania; ^3^ Department of Epidemiology, University of Medicine, Pharmacy, Science and Technology “George Emil Palade” of Târgu-Mureș, Târgu-Mureș, Romania; ^4^ Faculty of Pharmacy, University of Medicine, Pharmacy, Science and Technology “George Emil Palade” of Târgu-Mureș, Târgu-Mureș, Romania

**Keywords:** vascular surgery, COVID-19, pandemic, surgical activity, public health

## Abstract

The COVID-19 outbreak has placed substantial pressure on the medical systems worldwide. This study aimed to investigate the influence of the prepandemic vs. pandemic period on the activity of the Vascular Surgery Unit of a large emergency hospital in Eastern Europe. We performed a retrospective review of the vascular surgery cases admitted, comparing the statistics from the two time periods. We examined data of a total of 1,693 patients over the two periods. We report a 34.51% decrease in the surgical procedures performed during the pandemic period, with a disproportionate 80.6% decrease in the number of cases admitted with a diagnosis of venous insufficiency diagnosis and an increase of 67.21% in the number of patients admitted with acute arterial ischemia. Furthermore, individuals not classed as emergencies were delayed or denied surgical care. The number of nonurgent procedures conducted by our Vascular Surgery Unit decreased significantly, whereas the number of emergency surgeries increased. COVID-19′s effect is projected to have a long-term impact on how surgical treatments are provided in Romania.

## Introduction

**Figure 1:**
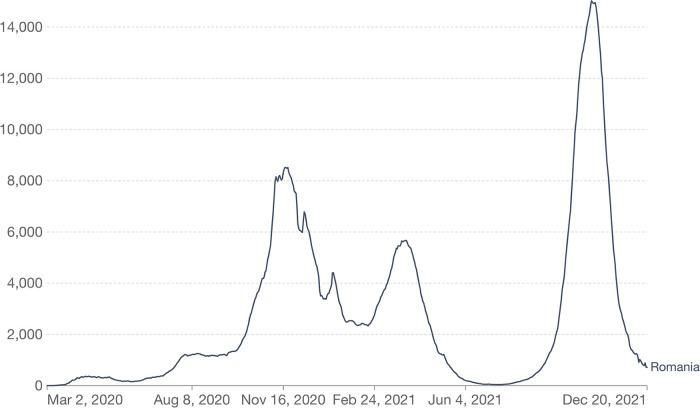
Evolution of the number of patients newly diagnosed with COVID-19 in Romania (6).

The declared epidemic of the severe acute respiratory syndrome-coronavirus-2 (SARS-CoV-2), also known as COVID-19, has disrupted the normal operations of the global healthcare system (1–3), with limits imposed in places with high infection rates. On 26 February 2020, the first verified COVID-19 case in Romania was documented (4). It was characterized by three waves of infection, from 26 February 2020 to 30 November 2021—the first maximum number of cases in 24 h was on 18 November 2020 (10,269), the second wave took place in March–June 2021, with a maximum number of infected patients in 24 h recorded on 25 March 2021 (6,651). The third wave occurred in September–November 2021, with a maximum number of 18,863 patients registered on 18 October 2021 (4). At the time when this article was written, there were 1,786,036 cases of COVID-19 in Romania and 57,099 fatalities (5) (Figure 1).

Once the number of cases began to rise, certain restrictions also affected medical activity, such as reducing the number of surgical procedures, delaying programmed operations, and deciding to admit emergencies only (7, 8). It was globally estimated that more than 28 million surgical interventions were rescheduled during lockdown (9). Delaying or canceling so many interventions can have a devastating impact on the medical system, patient prognosis, and patients’ quality of life.

Numerous publications revealed the effects of the pandemic on the activity of specific clinics dealing mainly with chronic pathologies, such as head and neck surgery, otorhinolaryngology, bariatric surgery, orthopedic surgery, cardiac surgery, plastic surgery, cardiology units, nephrology units, oncological clinics, and special transplant facilities (10–23).

The Vascular Surgery Unit of The County Hospital Târgu-Mureș is the only one of this type in the county. It also provides specific vascular emergency care for five other counties. The leading chronic pathologies admitted to the clinic are peripheral artery disease (PAD), chronic venous insufficiency (CVI), symptomatic carotid stenosis, and patients with end-stage chronic kidney disease for arteriovenous fistula (ESKD). The acute pathologies include acute ischemia of the upper and lower limb, arterial trauma, and ruptured aortic aneurysm (24–32).

As for many other departments in the hospital, the pandemic took a toll on the care provided to the patients. The characteristics of the procedures performed changed, as did the addressability, and the overall structure of the activity shifted toward supporting the emergency system and prioritizing acute strategies vs. elective surgery.

This study aims to compare the pandemic interval 1 June 2020 to 30 November 2021 with the prepandemic period of 1 June 2018 to 30 November 2019 and analyze the effects of the pandemic on vascular surgery activity.

## Materials and methods

This is a retrospective study analyzing the pandemic effect on the surgical activity of the Vascular Surgery Unit operating inside the Emergency County Hospital Târgu-Mureș, Romania, by comparing the pandemic period 1 June 2020 to 30 November 2021 with the same prepandemic time frame 1 June 2018 to 30 November 2019. The Vascular Surgery Unit was found in 2019, covering the territory of 250 km and a population of approximately 1.7 million people in Central Romania.

The number of patients admitted over the two time periods was selected from the computerized database of the Emergency County Hospital Târgu-Mureș by age, gender, diagnosis, type of surgical operation, and the number of hospitalization days.

Throughout the pandemic course, the unit was involved in emergency cases of both COVID-19 and non-COVID-19 patients.

Our cohort included PAD patients of any Leriche–Fontaine stage (33) who were candidates for surgical revascularization, endovascular revascularization, or major amputation, patients diagnosed with ESKD needing an arteriovenous fistula (AVF) for dialysis, patients having a history of cerebral ischemia and symptomatic carotid stenosis who underwent carotid thromboendarterectomy, patients with CVI who needed stripping or radiofrequency ablation (RFA) procedures, patients presenting with acute ischemia of the limbs solvable *via* Fogarty thrombectomy/embolectomy, and acute arterial trauma patients. Due to fewer performed procedures, patients undergoing lumbar sympathectomy, post-traumatic reconstructions, and excision of infected prosthetic grafts were grouped under the label “other procedures.” We excluded from the study candidates for extra-anatomical procedures, outpatients, patients treated conservatory, recipients of minor amputations (e.g., toe amputations), and patients presenting with acute limb ischemia solved by thrombolysis or mechanical thrombectomy, as these procedures are not performed in the unit. Also, the abdominal aortic aneurysm patients were excluded due to the small number of open repair procedures performed in the unit.

This study was conducted according to the guidelines of the Declaration of Helsinki and approved by the Institutional Ethics Committee of Târgu-Mureș Emergency County Hospital (no. Ad. 31750/7 December 2021).

Data are presented as the mean ± SD if normally distributed and median (Interquartile range) if nonparametrically distributed. Differences between groups were tested using a two-tailed Student's *t*-test or Mann–Whitney *U*-test as appropriate for two-group comparisons. Categorical variables were compared with the *χ*^2^-test. All *p*-values are two-tailed, with *p* < 0.05 considered statistically significant. Statistical analysis was performed using SPSS for Windows version 22.0 (SPSS, Inc., Chicago, IL, USA).

## Results

Among the surgical procedures performed in the Vascular Surgery Unit are saphenous stripping or RFA, vascular access procedures, infrainguinal and aortoiliac revascularization, carotid surgery, major amputations, endovascular treatment of various stenosis, and Fogarty thromboendarterectomy for acute limb ischemia.

A number of 1,693 patients were admitted to our clinic over the two periods, with a decrease of 34.51% in the performed surgical procedures during the pandemic period (1,023 patients were admitted in the prepandemic interval and 670 during the pandemic interval). However, there was a statistically increased number of ALI cases (*p* < 0.0001), followed by symptomatic carotid stenosis (*p* < 0.0001), STG IV Fontaine PAD patients (*p* < 0.001), and patients with CLI (*p* = 0.001). Moreover, no significant difference was observed between the two periods regarding ESKD patients (*p* = 0.66) and STG III PAD patients (*p* = 0.79). All other diagnoses had a significant decrease during the pandemic period, as shown in Table 1.

**Table 1:** Pathologies and basic characteristics for the prepandemic and the pandemic periods.

	Prepandemic 1 June 2018–30 November 2019	Pandemic 1 June 2020–30 November 2021	Difference (%)	*p* value
CVI	134 (13.1%)	26 (3.88%)	−9.22	<0.0001
ESKD	87 (8.5%)	61 (9.1%)	0.60	0.66
PAD	653 (63.83%)	342 (51.04%)	−12.79	<0.0001
STG II A Fontaine	114 (11.14%)	—	−11.14	0.0003
STG II B Fontaine	182 (17.79%)	57 (8.51%)	−9.28	<0.0001
STG III Fontaine	193 (18.87%)	123 (18.36%)	−0.51	0.79
STG IV Fontaine	164 (16.03%)	162 (24.18%)	8.15	<0.0001
Critical limb ischemia	357 (34.90%	285 (42.54%)	7.64	0.001
Symptomatic carotid stenosis	69 (6.74%)	100 (14.93%)	8.18	<0.0001
ALI	61 (5.96%)	102 (15.22%)	9.26	<0.0001
Other pathology	19 (1.86%)	39 (5.82%)	3.96	<0.0001
All patient	1023	670	−34.51	—
Mures-county no. (%)	579 (56.59%)	488 (72.83%)	—	0.0003^a^
Other county no. (%)	444 (43.41%)	182 (27.17%)	—	
Age mean ± SD, (min–max)	66.49 ± 11.03 (20–93)	66.47 ± 10.85 (19–98)	—	0.96^b^
Sex (M/F)	72.43%/27.57%	74.63%/25.37%	—	0.34^a^
Duration of hospital stay (days), median [interquartile range]	8 [4–12]	7 [4–11]	—	0.07^c^

CVI, chronic venous insufficiency; ESKD, end-stage kidney disease; PAD, peripheral arterial disease; STG, stage; ALI, acute limb ischemia; SD, standard deviation.

^a^ Chi-square test.

^b^ Student’s *t*-test.

^c^ Mann–Whitney test.

The addressability was analyzed based on the residential county of the patients. Although we recorded decreases in the numbers of patients referred to us both from the local Mures county and from neighboring counties, addressability from the neighboring counties decreased proportionally more. We recorded statistically significant differences between the prepandemic and pandemic periods, with a percentual increase of patients from the local county (56.59% vs. 72.83%, *p* = 0.0003) and a decrease of patients from other counties during the pandemic (43.41% vs. 27.17%. *p* = 0.0003).

There were no statistically significant differences between the two periods in terms of average age (66.49 ± 11.04 vs. 66.47 ± 10.85, *p* = 0.96), gender distribution (72.43%/27.57% vs. 74.63%/25.37%, *p* = 0.34), or hospitalization day (median days [interquartile range], 8 [4–12] vs. 7 [4–11], *p* = 0.07) (Table 1).

During the pandemic period, 142 COVID-19 patients were admitted to our Vascular Surgery Unit. The pathologies, types of surgeries, mean ages, gender distributions, hospitalization days, intensive care unit (ICU) admissions, and mortality rates are presented in Table 2. In terms of pathologies and types of surgeries, we registered a significant increase in acute limb ischemia (*p* < 0.0001) and amputation (*p* < 0.0001) in COVID-19 patients. Moreover, a substantial increase in the number of hospitalization days (*p* = 0.0001), ICU admission (*p* < 0.0001), and deaths (*p* < 0.0001) was detected in COVID-19 patients. Furthermore, in terms of gender distribution, significantly more women were admitted during the pandemic period (*p* < 0.0001) (Table 2).

**Table 2:** Characteristics of pandemic period patients.

Variables	Non-COVID-19 patients (*n* = 528)	COVID-19 patients (*n* = 142)	*p*-Value
CVI	26 (4.92%)	—	0.058^a^
ESKD	61 (11.55%)	—	0.01^a^
Symptomatic carotid stenosis	79 (14.96%)	21 (14.78%)	0.95^a^
Acute limb ischemia	58 (10.98%)	44 (30.98%)	<0.0001^a^
Other pathology	30 (5.68%)	9 (6.33%)	0.76^a^
Peripheral arterial disease			
Suprainguinal revascularization	70 (13.25%)	18 (12.67%)	0.85^a^
Infrainguinal revascularization	103 (19.50%)	23 (16.19%)	0.37^a^
Amputation	54 (10.22%)	36 (25.35%)	<0.0001^a^
Endovascular treatment	38 (7.19%)	—	0.02^a^
Age mean ± SD (min–max)	65.75 ± 12.17 (19–98)	67.19 ± 11.46 (36–84)	0.33^b^
Sex (M/F)	79.35%/20.65%	57.04%/42.96%	<0.0001^a^
Duration of hospital stay (days), median [interquartile range]	6 [4–9]	8 [7–11]	0.001^c^
ICU admission, no. (%)	135 (25.56%)	68 (47.88%)	<0.0001^a^
Deaths, no. (%)	77 (14.58%)	45 (31.69%)	<0.0001^a^

CVI, chronic venous insufficiency; ESKD, end-stage kidney disease; ICU, intensive care unit; SD, standard deviation.

^a^ Chi-square test.

^b^ Student’s *t*-test.

^c^ Mann–Whitney test.

The statistics show no significant differences in the number of surgical procedures performed in the two periods. The only procedure that has grown tremendously is carotid thrombendarteriectomy (*p* < 0.0001). In terms of the average age, only patients with symptomatic carotid stenosis (*p* < 0.0001) and patients who benefited from endovascular treatments (*p* < 0.0001) have been reported to be younger during the pandemic period. Fewer days of hospitalization have been recorded for carotid thrombendarteriectomy (*p* < 0.0001), upper and lower limb embolectomy (*p* = 0.01), suprainguinal revascularization (*p* < 0.0001), infrainguinal revascularization (*p* < 0.0001), and endovascular treatment (*p* < 0.0001) during the pandemic period. However, the number of days spent in the hospital was higher regarding amputations (*p* = 0.001), as shown in Tables 3, 4.

**Table 3:** Types of surgeries and their characteristics for both periods—part I.

	Prepandemic 1 June 2018–30 November 2019	Pandemic 1 June 2020–30 November 2021	Difference (%)	*p* Value
Chronic venous insufficiency				
Venous stripping	96 (71.64%)	21 (80.76%)	9.12	0.34
Radiofrequency ablation	38 (28.36%)	5 (19.24%)	−9.12	
Age mean ± SD (min–max)	55.14 ± 13.95 (20–83)	55.26 ± 16.98 (19–75)	—	0.97^a^
Sex (M/F)	43.28%/56.72%	38.46%/61.54%	—	0.81^b^
Duration of hospital stay (days), median [interquartile range]	3 [2–4]	3 [2.25–4]	—	0.36^c^
End-stage kidney disease				
rc-avf	41 (47.12%)	29 (47.54%)	0.42	0.96
bc-avf	30 (34.48%)	22 (36.06%)	1.58	0.84
bb-avf	16 (6.89%)	10 (16.39%)	9.5	0.75
Age mean ± SD (min–max)	67.5 ± 10.53 (36–89)	65.16 ± 13.53 (31–91)	—	0.26^a^
Sex (M/F)	66.67%/33.33%	63.93%/36.07%	—	0.54^b^
Duration of hospital stay (days), median [interquartile range]	2 [2–2]	2 [2–2]	—	0.27^c^
Symptomatic carotid stenosis				
Carotid endarterectomy	69 (6.74%)	100 (14.93%)	8.18	<0.0001
Age mean ± SD (min–max)	69.69 ± 7.58 (58–83)	65.65 ± 8.87 (37–82)	—	<0.001^a^
Sex (M/F)	63.76%/36.24%	70%/30%	—	0.49^b^
Duration of hospital stay (days), median [interquartile range]	5 [4–7]	4 [3–4]	—	<0.0001^c^
Acute limb ischemia				
Upper limb embolectomy	14 (22.95%)	32 (31.37%)	8.42	0.24
Lower limb embolectomy	47 (77.05%)	70 (68.63%)	−8.42	
Age mean ± SD (min–max)	67.22 ± 7.06 (54–88)	68.53 ± 12.21 (31–91)	—	0.38^a^
Sex (M/F)	83.61%/16.39%	74.51%/25.49%	—	0.24^b^
Duration of hospital stay (days), median [interquartile range]	11 [8–14]	8 [5–12]	—	0.01^c^

RC-AVF, radial-cephalic arteriovenous fistula; BC-AVF, brachial-cephalic arteriovenous fistula; BB-AVF, brachio-basilic arteriovenous fistula; SD, standard deviation.

^a^ Student’s *t*-test.

^b^ Chi-square test.

^c^ Mann–Whitney test.

**Table 4:** Types of surgeries and their characteristics for both periods—part II.

	Prepandemic 1 June 2018–30 November 2019	Pandemic 1 June 2020–30 November 2021	Difference (%)	*p* Value
Suprainguinal revascularization				
Ilio-femoral by-pass	52 (32.91%)	32 (36.36%)	3.45	0.60
Aorto-femoral by-pass	50 (31.65%)	32 (36.36%)	4.72	0.45
Aorto-bifemoral by-pass	56 (35.44%)	24 (27.27%)	−8.17	0.19
Age mean ± SD (min–max)	67.14 ± 8.54 (45–87)	65.59 ± 7.7 (47–82)	—	0.14^a^
Sex (M/F)	79.74%/20.26%	81.81%/18.19%	—	0.82^b^
Duration of hospital stay (days), median [interquartile range]	11.5 [8–14]	9 [7–11.5]	—	<0.0001^c^
Infrainguinal revascularization				
FP bypass with saphenous vein	28 (8.78%)	19 (15.07%)	6.30	0.054
FP bypass with grafts	230 (72.10%)	90 (71.43%)	−0.67	−0.88
Remote endarterectomy	61 (19.12%)	17 (13.49%)	−5.63	0.16
Age mean ± SD (min–max)	68.77 ± 8.95 (30–93)	68.13 ± 8.63 (36–91)	—	0.48^a^
Sex (M/F)	81.81%/18.19%	85.71%/14.29%	—	0.37^b^
Duration of hospital stay (days), median [interquartile range]	10 [7–14]	7 [6–10]	—	<0.0001^c^
Amputation				
AKA	33 (53.23%)	47 (52.23%)	−1	0.90
BKA	29 (46.77%)	43 (47.77%)	1	
Age mean ± SD (min–max)	68.19 ± 9.05 (36–91)	70.71 ± 9.75 (47–91)	—	0.1^a^
Sex (M/F)	77.41%/22.59%	76.66%/23.34%	—	0.92^b^
Duration of hospital stay (days), median [interquartile range]	8.5 [6–11]	11.5 [7–11]	—	0.001^c^
Endovascular treatment				
Balloon angioplasty	74 (64.91%)	21 (55.26%)	−9.65	0.28
Stenting	40 (35.09%)	17 (44.74%)	9.65	
Age mean ± SD (min–max)	66.42 ± 11.25 (32–88)	60.55 ± 15.46 (19–84)	—	0.03^a^
Sex (M/F)	77.19%/22.81%	63.15%/36.86%	—	0.13^b^
Duration of hospital stay (days), median [interquartile range]	9 [5–13]	5.5 [4–8]	—	<0.0001^c^

FP, femoral-popliteal; SD, standard deviation; AKA, above-knee amputation; BKA, below-knee amputation.

^a^ Student’s *t*-test.

^b^ Chi-square test.

^c^ Mann–Whitney test.

Furthermore, the number of in-patients admitted each month was analyzed. A significant decrease was registered during the pandemic, with one exception: in May, the same number of patients were admitted in both periods, in 2020–2021, during the pandemic, and in 2018–2019. The decreases in the number of patients were 76.19% in December 2020, compared to the corresponding month of 2018, followed by 73.91% in November 2021, compared to 2019, and 63.93%decrease in September 2021, compared to 2019 (Table 5).

**Table 5:** Hospital admissions/month pandemic/prepandemic.

	Prepandemic 2018	Pandemic 2020	Difference (%)	Pandemic peaks
June	73	56	−23.29	
July	64	53	−17.19	
August	65	50	−23.08	
September	67	51	−23.88	
October	54	49	−9.26	
November	66	28	−57.58	First peak
December	42	10	−76.19	
	2019	2021		
January	68	52	−23.53	
February	59	24	−59.32	
March	55	43	−21.82	Second peak
April	36	31	−13.89	
May	39	39	—	
June	58	40	−31	
July	48	34	−29.17	
August	48	38	−20.83	
September	61	22	−63.93	
October	51	32	−37.25	Third peak
November	69	18	−73.91	

January–December, the months of the year.

## Discussion

An overall decrease in surgical procedures was experienced throughout the pandemic, significantly affecting chronic pathologies such as CVI and the initial stages of PAD. Due to the alarming infection rate, lockdowns were established everywhere. Nationally, the first lockdown was initiated on 24 March 2020. All chronic admittances were canceled, and all surgical activity was redirected toward emergencies. Therefore, the Vascular Surgery Unit experienced a significant increase in the number of patients diagnosed with symptomatic carotid stenoses eligible for endarterectomy, patients with ALI of the upper and lower limb, and patients with ischemia-induced trophic disorders of such amplitude that a major amputation was required. We believe that the thrombotic effect of the virus may have influenced the more significant number of acute ischemia.

The number of patients presenting with lower stages of PAD and CVI decreased. Also, except for amputation patients, the hospitalization days were reduced for all categories of patients admitted to minimalize hospital contact and decrease the risk of infection with COVID-19.

Schuivens et al., in a paper on the effect of the pandemic on peripheral artery disease patients, recorded an increase of major amputations, with an incidence of 42% in 2020, compared with 18% in 2019 and 15% in 2018 (34). In a paper by Mascia et al. on the impact of the pandemic on the medical system in Italy, the March–April 2020 period was compared with the same months in 2019. There was an increase in acute ischemia cases (26.7% compared with 17.6%), critical ischemia (20.7% vs. 2.9%), and aortic pathology (35). Similar results to the study are described by Ilonzo et al., who found an increase to mainly affect the candidates for major amputations [37.7% (2020) vs. 15.2% (2019)] and the symptomatic carotid stenosis patients (9.6% vs. 4%) (36).

Miranda et al. analyzed the activity of the Vascular Surgery Clinics of nine tertiary hospitals, comparing the lockdown period 18 March–17 May and the immediately following period when the restrictions were withdrawn, 18 May–31 December, with the corresponding periods in 2019. The results were a decrease of the procedures by 64% compared to 2019, whereas in the following period, there was only a 14% decrease compared to 2019. According to their study, venous pathology recorded the highest decrease, followed by endovascular procedures and aortic aneurysm repair. The only procedure registering an increase in the number of interventions during the pandemic was amputation, which increased by 7% (37).

As shown in Table 2, the COVID-19 patients were associated with a higher risk of acute limb ischemia, major amputation, and death. Similar to our results, according to a meta-analysis conducted by Galyfos, SARS-CoV-2 infection is associated with a significant risk of thrombotic events, including ALI. COVID-related ALI is often caused by producing a systemic inflammatory response and a prothrombotic state but also in patients with a low comorbidity rate who have a high risk of mortality and amputation. Concerning the virus's thrombogenicity, numerous techniques for treating ALI, such as thrombembolectomy, thrombolysis, thrombosuction, and others, have been tried with comparable effectiveness in terms of limb salvage (38).

As the virus was spreading and the severe cases of SARS-CoV-2 were accumulating, Infectious Diseases, Pulmonary Diseases, and then Intensive Care Units were filling up, and organization measures were necessary. Some units were transformed into COVID-19 hospitals, taking care of this pathology alone. The Vascular Surgery Unit is the only of this kind in the county, so it had to provide care for both COVID-19 and non-COVID-19 pathologies. A whole wing was transformed into a COVID-19 facility in the County Hospital, with a dedicated COVID-19 Operating Room.

Apart from the lockdown periods, medical activity was continuously affected. New protocols were elaborated, and the 1.5–2 m distance between the patients in the wards had to be respected. The chronic, ambulatory patients who needed clinical follow-up were evaluated respecting two patients per hour regulations. These measures affected the waiting period and the regular flux schedule. The COVID-19 pandemic affected medical activity globally, causing a decrease in the addressability of chronic cases and increased referred emergencies.

As some of the tertiary units around the county were entirely transformed into COVID-19 facilities, a significant number of patients were addressed to nearby vascular surgery units. On the other hand, some of these patients with chronic pathologies were reluctant to travel to another county, leading to unavoidable delays in treatment. In the prepandemic period, the addressability from other counties to our vascular surgery unit was greater than that during the pandemic, explainable by the restrictions and limitations to travel and the redirecting of the cases to the nearest vascular center.

Another interfering issue worth mentioning is the staff redistribution: many trainées were reassigned to the COVID-19 units to help general practice, making it harder to maintain the same rhythm and disrupting their vascular surgical training.

The strong point of this paper is the study of the whole pandemic course. We analyzed its effect on the number of patients and the hospitalization days for each pathology and procedure in our service. The limitations of the study are the relatively small number of patients from a single center, mainly men, making extrapolation difficult. Multicentric, retrospective studies are required for better accuracy of the results and a more extensive overview of the national impact of the pandemic on the healthcare system.

## Conclusions

The SARS-CoV-2 pandemic was a worldwide public health problem affecting all areas of medical interest. Our Vascular Surgery Unit was also influenced by the pandemic changes in the healthcare system, suffering a significant decrease in the number of performed non-urgent procedures and increasing the number of emergencies attended. It is questionable whether the increase in the number of amputations during the pandemic was due to the forceful delaying of the chronic pathology or simply because the addressability of this type of emergency has grown.

## Data Availability

The raw data supporting the conclusions of this article will be made available by the authors, without undue reservation.
